# Preparation and clinical evaluation of a novel lozenge containing polaprezinc, a zinc-L-carnosine, for prevention of oral mucositis in patients with hematological cancer who received high-dose chemotherapy

**DOI:** 10.1007/s12032-016-0795-z

**Published:** 2016-07-14

**Authors:** Hiroko Hayashi, Ryo Kobayashi, Akio Suzuki, Yuto Yamada, Masayuki Ishida, Toshinobu Shakui, Junichi Kitagawa, Hideki Hayashi, Tadashi Sugiyama, Hirofumi Takeuchi, Hisashi Tsurumi, Yoshinori Itoh

**Affiliations:** 1 Department of Pharmacy, Gifu University Hospital, 1-1 Yanagido, Gifu, 501-1194 Japan; 2 Laboratory of Pharmacy Practice and Social Science, Gifu Pharmaceutical University, Gifu, Japan; 3 First Department of Internal Medicine, Gifu University Graduate School of Medicine, Gifu, Japan; 4 Laboratory of Pharmaceutical Engineering, Gifu Pharmaceutical University, Gifu, Japan

**Keywords:** Polaprezinc, Lozenge, Oral mucositis, Analgesic, High-dose chemotherapy, Hematopoietic stem cell transplantation

## Abstract

We previously reported that oral ingestion of polaprezinc, a zinc-L-carnosine, suspended in sodium alginate solution prevents oral mucositis in patients receiving radiotherapy or high-dose chemotherapy. In the present study, we developed a novel preparation of polaprezinc and evaluated clinical effect of the lozenge preparation in patients receiving high-dose chemotherapy for hematopoietic stem cell transplantation. The preparation contained 18.75 mg polaprezinc in a tablet and showed an excellent uniformity and stability up to 24 weeks after storage under room temperature. The incidence rate of grade ≥ 2 oral mucositis was 74 % in patients without premedication, whereas the rate was remarkably reduced in patients receiving the suspension (23 %) or lozenge (13 %) of polaprezinc (*P* < 0.01). The use of non-opioid analgesic drugs such as anti-inflammatory agents and local anesthetics for oral pain was also greatly reduced by polaprezinc suspension or its lozenge (16 % for suspension and 13 % for lozenge compared with 89 % with no premedication, *P* < 0.01). These findings suggest that polaprezinc lozenge is simple to apply and highly effective for prevention of oral mucositis associated with high-dose chemotherapy for hematopoietic stem cell transplantation.

## Introduction

Oral mucositis is one of the most common and debilitating complication of cancer treatment, particularly chemotherapy and radiotherapy. This adverse reaction occurs in 20–40 % of patients receiving conventional chemotherapy, in 80 % of patients receiving the conditioning high-dose chemotherapy for hematopoietic stem cell transplant (HSCT), and in almost all patients with head and neck cancer receiving radiotherapy [1–3]. Oral mucositis is often accompanied by pain, odynophagia, dysgeusia and subsequent dehydration and malnutrition, which reduces patients’ quality of life (QOL) [4, 5]. In severe case, discontinuation of therapy or dose reduction is required, which results in the decrease in dose intensity of therapy and reduction in the therapeutic effect [6, 7]. In addition, the incidence of oral mucositis has negative impact on the healthcare economy requiring costs of care associated with hospitalization, medical management, nutritional support, and management of secondary infection [6, 8]. We also reported that oral mucositis is a significant risk of prolongation of hospital stay in patients with head and neck cancer who received radiotherapy alone or in combination with chemotherapy [9].

Although the precise mechanisms underlying the cytotoxic action of chemotherapy or/and radiotherapy for induction of oral mucosa remain to be clarified, the development and healing of oral mucositis are characterized by the following processes: (1) initiation of mucosal injury due to the production of reactive oxygen species, (2) tissue injury and cell death induced by upregulation of pro-inflammatory cytokines such as tumor necrosis factor (TNF)-α, interleukin (IL)-1 and IL-6, (3) formation of mucosal ulceration caused by infiltration of inflammatory cells and worsening of symptoms due to secondary infection, and (4) healing by epithelial proliferation as well as cellular and tissue differentiation [10].

Several agents, including benzydamine [11, 12], sucralfate [13], prostaglandin E2 [14], glutamine [15], granulocyte–macrophage colony-stimulating factor [16], palifermin [17–19], and amifostine [20], have been reported for prevention of oral mucositis; however, most of them, except for palifermin, have no consistent effect. We recently reported that oral ingestion of polaprezinc, a zinc-L-carnosine, suspended in sodium alginate solution was highly effective for prevention of oral mucositis associated with radiotherapy in patients with head and neck cancer as well as in patients with high-dose chemotherapy for HSCT [21, 22]. However, there were several drawbacks in the preparation: (1) preparation of polaprezinc suspension is time-consuming, (2) the ingredient is rapidly separated from sodium alginate solution, (3) dosing is not accurate due to the high viscosity of the suspension, and (4) oral acceptability is limited in some patients due to unfavorable taste and undesirable texture. To overcome these problems of the suspension, we have developed a polaprezinc lozenge and evaluated the clinical effect of the preparation for prevention of oral mucositis in patients who received conditioning high-dose chemotherapy for HSCT.

## Patients and methods

### Materials

Polaprezinc (Promac^®^ granules 15 %) was purchased from Zeria Pharmaceutical Co. Ltd. (Tokyo, Japan). Sodium alginate (KIMICA Algin I-1^®^, Kimica Co. Ltd., Osaka, Japan), magnesium Stearate (Magnesium Stearate^®^, Mallinckrodt Japan Co. Ltd., Tokyo), acesulfame potassium (Sunett^®^ Pharma Grade Type I, MC Food Specialties Inc., Tokyo), aspartame (Aspartame^®^, Ajinomoto Co., Inc., Tokyo), mannitol (Parteck M100^®^, Merck Ltd., Tokyo), microcrystalline cellulose (CEOLUS UF-711^®^, Asahi Kasei chemicals Corp., Tokyo), cornstarch (PC-10 ^®^, Asahi Kasei chemicals Corp., Tokyo), fragrance material (dry coat^®^, Takata Koryo CO., LTD., Hyogo, Japan) were obtained from commercial sources and used as base materials of the lozenge preparation.

### Preparation of polaprezinc lozenge for oral application

The compositions of polaprezinc lozenge are shown in Table 1. The mixture was directly compressed with 15 kN by using a single punch tablet press (TAB ALL N-30E Type ^®^, Okada Seiko Co. Ltd., Tokyo). One tablets of the lozenge contained 18.75 mg polaprezinc. The thickness and diameter of the preparation were 5.8 and 16.3 mm, respectively. For application of patients, one piece of polaprezinc lozenge was sucked and swallowed for 4 times in a day.

**Table 1:** Composition of polaprezinc lozenge in one tablet

Polaprezinc	18.75 mg
Sodium alginate	0.05 g
Magnesium stearate	0.005 g
Acesulfame potassium	0.0015 g
Aspartame	0.0015 g
Mannitol	0.33 g
Cellulose	0.4 g
Cornstarch	0.05 g
Fragrance material	0.01 g

### Preparation of polaprezinc sodium alginate suspension

Polaprezinc (75 mg) was suspended in 20 mL of 5 % sodium alginate solution. A 5 mL portion of the suspension was orally rinsed for 2 min and then swallowed for 4 times in a day and continued until 1 month after transplantation.

### Determination of polaprezinc

The content uniformity of the polaprezinc lozenge was tested in 10 separate preparations. Polaprezinc was determined by HPLC with spectrophotometric detection. Sample preparation for HPLC analysis was carried out by the following methods: the tablet was accurately weighted and finely pulverized, and the resultant powder was suspended in 10 mL of 0.1 M hydrochloric acid solution. Then, the suspension was centrifuged at 2000× *g* for 5 min. The supernatant was filtrated with 0.2-µm filter and used for analysis. A 10-μl portion of the filtrated supernatant was directly injected onto HPLC.

The HPLC system consisted of a separation column (Shim-pack FC-ODS; 150 mm × 4.6 mm in diameter, 5 μm of sphere size, Shimadzu, Kyoto, Japan), column oven whose temperature was set at 40 °C, and spectrophotometric detector (SPD-10A, Shimadzu). The mobile phases were 10 mM phosphate buffer at pH 3.5 (A) and acetonitrile (B). The HPLC analysis was carried out by using a gradient elution with the following method: the concentration of mobile phase B was started at 10 % from 0 to 2 min, then, the linear gradient from 10 to 50 % applied from 2 to 4 min and increased at 50 % from 4 to 6 min. Thereafter, the acetonitrile concentration was lowered to 10 % from 6 to 7 min and maintained the same composition for further 3 min. Polaprezinc was detected at 210 nm.

### Uniformity of polaprezinc lozenge preparation

The acceptance value (AV) of the preparation is less than 15 %, according the Japanese Pharmacopoeia 16th edition (JP16). AV for JP 16 was calculated according to the following equation: AV = | *M* *−* *X* | + *k* *s,* in which *M* is reference value, *X* is the average of individual contents expressed as the percentage of the label claim, *k* is acceptability constant, and *s* represents sample standard deviation.

### Stability test

A portion of the preparation was stored at 25 °C with 50–60 % humidity (normal condition) or at 40 °C with 75 % humidity (accelerated condition) for 2–24 weeks, and then the content of polaprezinc was determined. Another set of test was performed to determine the hardness of the stored preparation.

### Evaluation of clinical effect of polaprezinc lozenge for prevention of oral mucositis in patients with conditioning high-dose chemotherapy for HSCT

Patients were pretreated with either polaprezinc suspension during January 2013 and December 2014 (*N* = 31) or polaprezinc lozenge during January 2015 and December 2015 (*N* = 16) for prevention of oral mucositis. Data were compared between the two groups. Moreover, the data obtained from patients who received high-dose chemotherapy without any premedication during March 2006 and February 2011 were used as negative control.

The incidence and severity of oral mucositis and its associated symptoms such as oral pain and other adverse events were reviewed from medical records and compared among three groups. The severity of adverse events was graded according to the Common Terminology Criteria for Adverse Events (CTCAE) version 3.0. The prevalence of the use of non-opioid analgesics, including anti-inflammatory drugs and local anesthetics, and opioid analgesics was also compared.

### Ethical approval

This study was carried out in accordance with the guidelines for human studies adopted by the ethics committee of the Gifu University Graduate School of Medicine and notified by the Japanese government (Institutional review board approval No. 25–79). All participants except for patients who received high-dose chemotherapy without any premedication provided written informed consent prior to participation.

#### Statistical analysis

Data were analyzed by using IBM SPSS statistics version 22(IBM Japan Services Co., Ltd., Tokyo). The incidence rates of adverse events were statically compared among three groups by Kruskal–Wallis test followed by Dunn’s test or Scheffe’s test for multiple comparison. Parametric variables were analyzed by one-way analysis of variance followed by Scheffe’s test. *P* values of <0.05 were considered statically significant.

## Results

The average of polaprezinc content in 10 preparation of polaprezinc lozenge was 19.6 + 0.52 mg, and the values were ranging from 101.6 to 108.7 %. The relative standard deviation was 2.8 %. Thus, *AV* was 9.2 %, a value that was within the limit (15 %) of uniformity of dosage units for JP 16.

**Fig. 1:**
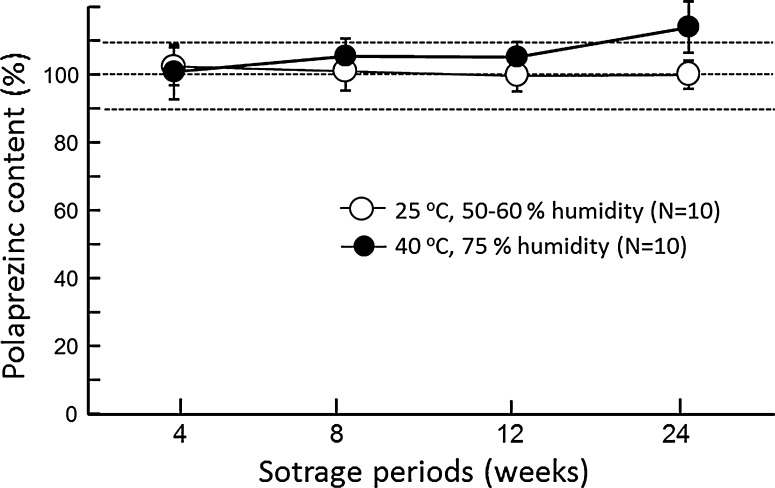
Stability of polaprezinc lozenge after storage under normal condition (A) or accelerated condition (B) for up to 24 weeks. Each tablet was wrapped in an aluminum package and stored at 25 °C with 50–60 % humidity (normal condition) or at 40 °C with 75 % humidity (accelerated condition). Each column represents the mean ± SD of 10 experiments

When the polaprezinc lozenge was stored in polyethylene package under normal condition for 24 weeks, no apparent changes in the polaprezinc content, form, or color of preparation were observed. The contents of polaprezinc were fairly stable ranging 99.8 % to 102.4 % during 24 weeks after storage at 25 °C with 50–60 % humidity (normal condition) (Fig. 1). However, a slight change in color from white to light brown appeared in the preparation stored for 24 weeks in a chamber controlled at 40 °C and 75 % in humidity (accelerated condition), although the contents of polaprezinc were almost stable (Fig. 1).

The demographics of patients receiving no premedication, premedication with either suspension or lozenge of polaprezinc are shown in Table 2. There were no significant differences in gender, age, laboratory data, type of leukemia and the rate of regimen containing Ara-C or MTX among three groups.

**Table 2:** Patient demographics and clinical characteristics

	No premedication	Polaprezinc suspension	Polaprezinc lozenge	*P* value
Number of patients (male/female)	19 (13/6)	31 (24/7)	16 (10/6)	0.755^a^
Age (medium, range)	49.2 (26–73)	54.5 (19–73)	55.8 (22–70)	0.370^b^
Serum albumin (g/dL)	3.9 ± 0.5	3.7 ± 0.5	3.9 ± 0.4	0.370^c^
Aspartate transaminase (U/L)	30.8 ± 28.6	28.3 ± 26.4	23.1 ± 9.5	0.643^c^
Alanine aminotransferase (U/L)	25.8 ± 15.5	31.6 ± 32.8	23.6 ± 18.3	0.543^c^
Serum creatinine (mg/dL)	0.87 ± 0.77	0.72 ± 0.19	0.62 ± 0.14	0.235^c^
White blood cells (/mm^3^)	5534 ± 5010	4162 ± 2928	4389 ± 3030	0.734^c^
Hemoglobin (g/dL)	10.5 ± 2.3	10.1 ± 2.0	10.8 ± 2.4	0.549^c^
Platelet (/mm^3^)	15.4 ± 7.1	17.1 ± 14.4	20.4 ± 14.9	0.515^c^
Diagnosis (%)				0.487^a^
Acute myeloid leukemia	1 (5.3)	10 (32.3)	5 (31.3)	
Acute lymphoid leukemia	8 (42.1)	1 (3.2)	3 (18.8)	
Acute promyelocytic leukemia	0	1 (3.2)	0	
Myelodysplastic syndromes	1 (5.3)	2 (6.4)	0	
NK/T cell lymphoma		7 (22.6)	2 (12.5)	
Diffuse large B cell lymphoma	5 (26.3)	8 (25.8)	5 (31.3)	
Mantle cell lymphoma	1 (5.3)	0	1 (6.3)	
Follicular lymphoma	0	1 (3.2)	0	
Hodgkin lymphoma	1 (5.3)	1 (3.2)	0	
Chemotherapy regimen (%)				0.730^a^
Ara-C-based regimen	7 (38.9)	16 (51.6)	10 (62.5)	
MTX-based regimen	11 (61.1)	15 (35.5)	6 (25.0)	

^a^ *m* × *n* Chi-square test, ^b^ Kruskal–Wallis test, ^c^ one-way ANOVA followed by Scheffe test

**Fig. 2:**
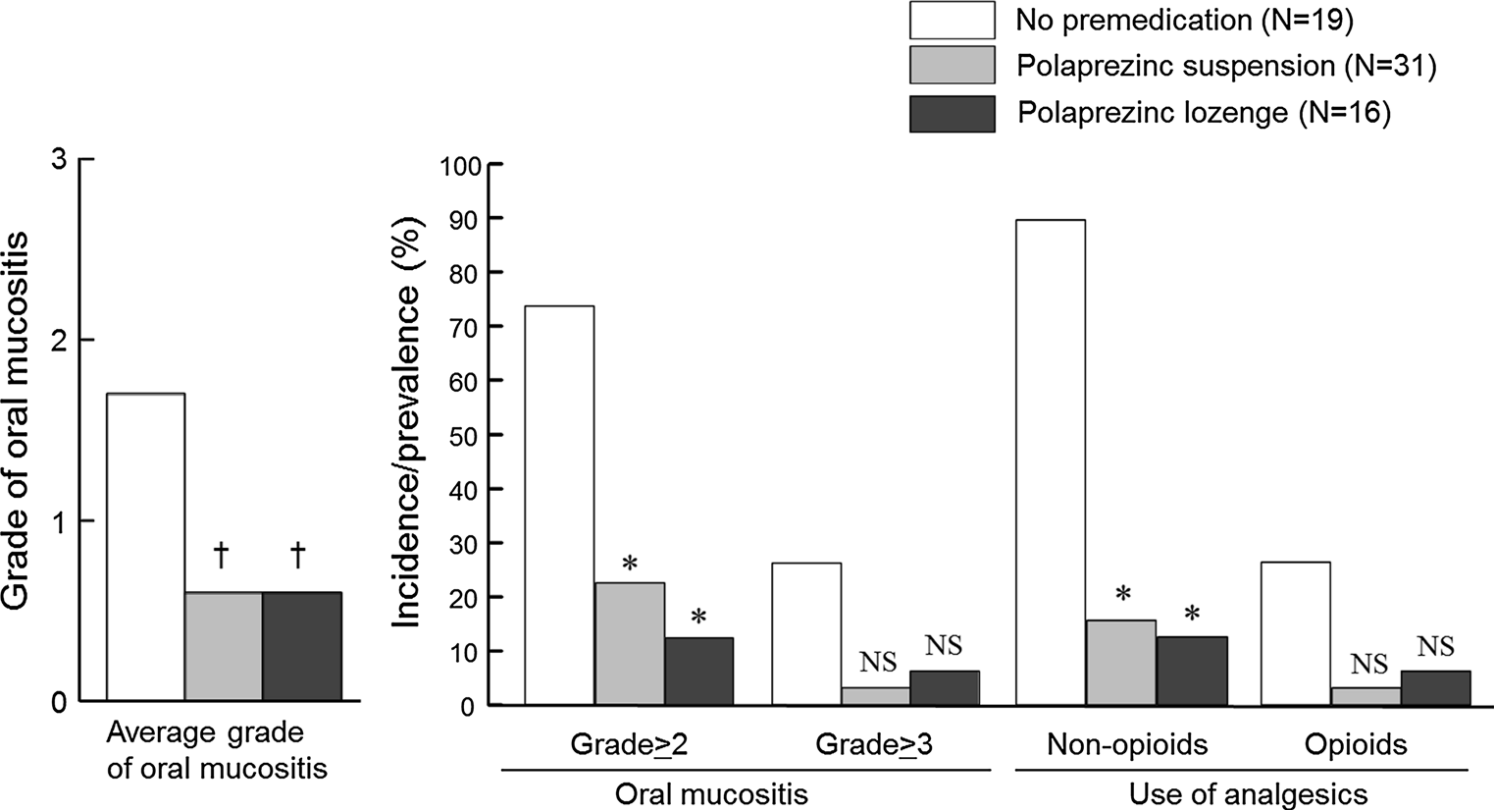
Comparison of effects of polaprezinc suspension and lozenge on the incidence and grade of oral mucositis and prevalence of the use of analgesics in patients receiving conditioning high-dose chemotherapy for stem cell hematopoietic stem cell transplantation. †*P* < 0.01 versus control by Kruscal-Wallis test followed by Dunn’s test, *P < 0.01 versus control by Kruscal-Wallis followed by Scheffe’s test. No significant difference in each parameter was observed between suspension and lozeng

The polaprezinc lozenge preparation stored at normal condition for up to 12 weeks was used in the present clinical study. As shown in Fig. 2, the incidence rates of grade 2 and grade 3 oral mucositis were 73.7 and 21.1 %, respectively, in no premedication control group, while the rates were greatly reduced in polaprezinc suspension group (22.6 % for grade 2, *P* < 0.01 by Kruskal–Wallis test followed by Scheffe’s test, and 3.2 % for grade 3, NS) and polaprezinc lozenge group (12.5 % for grade 2, *P* < 0.01, and 6.3 % for grade 3, NS). The average grade of oral mucositis was 0.6 for suspension group as well as for lozenge group, both of which were significantly lower than that (1.7) for control group (*P* < 0.01 by Kruskal–Wallis test followed by Dunn’s test). On the other hand, there was no significant difference in the average grade or incidence rate of oral mucositis between suspension group and lozenge group.

Similarly, the incidence rates of moderate to severe pain were also markedly reduced in suspension group and lozenge group as compared with control group. As a consequence, the prevalence for the use of non-opioid analgesics markedly decreased in suspension group (16.1 %, *P* < 0.01, for suspension group; 12.5 %, *P* < 0.01, for lozenge group), compared with control group (89.5 %), although the prevalence for the use of opioid analgesics was not significantly different among three groups.

As shown in Table 3, there were no significant differences in the incidence rates of other adverse events such as rash, pruritus, erythema, nausea, vomiting, and febrile neutropenia among there groups.

**Table 3:** Comparison of the incidence of other non-hematological adverse events among patients receiving non-premedication, suspension and lozenge preparations of polaprezinc

	No premedication	Polaprezinc suspension	Polaprezinc lozenge	*P* value
Rash	6 (31.6)	13 (41.9)	4 (25.0)	0.109
Pruritus	6 (31.6)	8 (25.8)	3 (18.7)	0.875
Erythema	6 (31.6)	11 (35.5)	6 (37.5)	0.991
Nausea (grade > 2)	6 (31.6)	12 (38.7)	13 (81.3)	0.493
Vomiting	6 (31.6)	3 (9.7)	2 (12.6)	0.257
Febrile neutropenia	18 (94.7)	25 (80.6)	13 (81.3)	0.624

Kruskal–Wallis test followed by Scheffe test

## Discussion

The present newly developed polaprezinc lozenge met the criteria of uniformity defined as <15 % of AV in the dosage uniformity by JP 16. This preparation was stable in the content of polaprezinc, when stored even under accelerated condition (40 °C, 75 % in humidity) for up to 12 weeks. However, slight change in color of the preparation was observed at 24 weeks, only when stored under accelerated condition. In the present study, the lozenge preparation stored within 12 weeks under normal condition was used for clinical study.

In the present clinical study, polaprezinc suspension markedly reduced the incidence of moderate to severe oral mucositis, as reported earlier [22]. The polaprezinc lozenge was confirmed to be as effective as the suspension for prevention of oral mucositis associated with high-dose chemotherapy. The accompanying symptom such as oral pain was also remarkably reduced by polaprezinc lozenge as well as the suspension.

On the other hand, there were no significant differences in the rates of other adverse events such as rash, pruritus, erythema, nausea, vomiting, and febrile neutropenia among three (control, suspension, and lozenge groups). These data suggested that there was no marked difference in the intensity of the chemotherapy.

Although the mechanisms of chemotherapy-induced oral mucositis remain to be clarified, production of reactive oxygen species and proinflammatory cytokines such as TNF-α and IL-6 in the oral mucosa after exposure to high-dose chemotherapy is considered to be implicated in the pathogenesis [23, 24]. Polaprezinc is currently used as anti-ulcer drug. It contains zinc, an essential trace element used for the therapy of gastric ulcer [25], in its molecule. It has been demonstrated that polaprezinc has protective action on the mucosal cells against noxious stimuli [26].The anti-oxidant action is considered to contribute to the mucoprotective action of polaprezinc [27, 28]. It has also been demonstrated that the mucoprotective action of polaprezinc is mediated at least in part by the enhancement of the expression of hemeoxygenase (HO)-1 [29]. Naito et al. [30] reported that polaprezinc reverses aspirin-induced increases in lipid peroxidation, neutrophil accumulation, and TNF-α expression in rat gastric mucosa. On the other hand, Wada et al. reported that enhancement of the expression of 72-kDa heat shock protein, an endogenous cytoprotective factor, plays an important role in the mucoprotective action of polaprezinc [31]. Therefore, it is highly likely that the antioxidative action of polaprezinc may contribute to the preventive effect of this compound against oral mucositis.

The clinical practice guidelines for the management of mucositis secondary to cancer therapy promulgated by the Multinational Association of Supportive Care in Cancer (MASCC)/International Society of Oral Oncology (ISOO) suggest that oral ingestion of zinc supplements may be of benefit to prevent oral mucositis in patients receiving radiotherapy or chemotherapy [23].

Currently, cancer chemotherapy is shifting from inpatient admission to outpatients setting. However, some of cytotoxic anti-cancer drugs or molecularly targeted drugs for the treatment of cancer in application to outpatients frequently develop an oral mucositis. Polaprezinc lozenge is a solid portable dosage form and can be easily and safely taken without water. Thus, it is highly probable that the present lozenge preparation is applicable not only to hospitalized patients but also to those in the ambulatory chemotherapy setting.

In conclusion, we newly developed the lozenge containing polaprezinc for prevention of oral mucositis. The preparation showed an excellent uniformity and stability. The polaprezinc lozenge was highly effective for prevention of moderate to severe oral mucositis in patients receiving high-dose chemotherapy for HSCT. The efficacy of the lozenge preparation was almost comparable to that of polaprezinc suspension in sodium alginate. Both the lozenge and suspension also reduced the occurrence of accompanying oral pain to the similar extent. Therefore, it is suggested that the present polaprezinc lozenge preparation is potentially useful for prevention of oral mucositis in cancer patients who receive high-dose chemotherapy.
